# Pattern and Outcomes of Injuries among Trauma Patients in Gedeo Zone, Dilla, South Ethiopia: A 5 Years Retrospective Analysis

**DOI:** 10.4314/ejhs.v30i5.14

**Published:** 2020-09

**Authors:** Bedru Jemal Abafita, Semagn Mekonnen Abate, Hilemariam Mulugeta Kasim, Bivash Basu

**Affiliations:** 1 Dilla University, college of Health Sciences and medicine, Department of Anesthesiology, Dilla, Ethiopia; 2 University of Calcutta, medical college, department of Anesthesiology, India

**Keywords:** Injury, Epidemiology, Outcome, Ethiopia

## Abstract

**Background:**

Injury has become a life threatening community health problem associated with significant mortality and morbidity worldwide. The aim of this study was to assess the burden of injury in Dilla University Hospital.

**Methods:**

Institution-based retrospective cross-sectional study was conducted from January 2015 to June 2019. Data was collected using questionnaire adapted from WHO injury surveillance guideline. Bivariate and multivariate logistic regressions were performed to determine the factors associated with hospital mortality.

**Results:**

Road traffic accident was the commonest cause of injury 178(47.3%) followed by interpersonal violence 113(30.1%). Revised trauma score (RTS) < 10 (AOR=2.5; 95% CI, 1.8–25.6), Glasgow coma scale (GCS) (AOR =0.3; 95% CI, 0.13–0.5), length of hospitalization (LOS) 1–7 days (AOR=0.1; 95% CI, 0.01–0.8) and time of arrival >24hr were predictors of mortality in a patient with injury.

**Conclusion:**

Lower extremity injury was common and mostly associated with RTA. Pre-hospital emergency medical service system and trauma registry need to be established to decrease the burden of injury.

## Introduction

Injury has become a life-threatening community health problem associated with significant mortality and morbidity worldwide (1,2,11–18,3–10). According to the World Health Organization (WHO) injury and violence surveillance, more than 5 million people die per year due to injury which accounts for 9% of the world's deaths (19). This figure is more than the combined fatalities resulting from human immunodeficiency virus (HIV/AIDS), malaria and tuberculosis. Approximately, 90% of injury related mortality occurred in low and middle income countries. Road traffic injuries are one of the leading causes of death which accounted for a quarter of 5 million injury deaths, specifically in the 15–29 age categories (19). It is predicted to be the seventh leading cause of death by 2030 in the world (19). In sub-Saharan Africa, injury related mortality and morbidity are very high specifically in low and middle income countries from which road traffic injury takes the lion's share (10,20,21). Recent Global Burden of Disease (GBD) showed that mortality related with injury in sub-Saharan Africa is estimated to be 14.6/100000 persons in 2020 compared to 97/100000 persons worldwide (21).

In Ethiopia, epidemiological studies showed that the pattern and outcomes of injury are variable in different regions of the country. A study conducted in the University of Gondar revealed that the prevalence of injury was 25%, and of these, 82% were young males. The commonest mechanisms of injury were assault (49.9%) and road traffic accidents (48%) (7). Another multicenter study conducted in Amhara regional state showed that the prevalence of injury in the region was 55.5% (6). Those who were young and daily laborers, substance abusers, and those who were had low monthly incomes were the most likely injury victims (6). A study conducted in Tikur Anbesa Specialized and Teaching Hospital showed that the prevalence of injury was 32.5%. In this cross-sectional study, road traffic accident was the most common mechanism of injury (38%) followed by violence (31.5%). Young population (20–29 years) and those with low monthly income (less than 650 Ethiopian Birr) were more likely to sustain injury incidents compared to the other population groups(22).

In Ethiopia, there is no national prevalence of trauma and national database injury registry for health planners and policymakers who are in need of the national prevalence of injury. Therefore, there is a need to have the prevalence and outcomes of trauma from different areas of the country for planning and management strategies of injury. The aim of this study is thus to assess the epidemiology and outcomes of injury in Gedeo zone.

## Methods

**Study area**: The study was conducted in Dilla University Teaching and Referral Hospital which is found in Dilla Town, Gedeo Zone, on the main road from Addis Ababa to Kenya, 360km South of Addis Ababa, 90km South of Hawassa (the capital of Southern Nations, Nationalities and People's Region (SNNPR). It is one of the public university hospitals; it provids health services to more than 4 million population of Gedeo Zone and neighboring catchment areas of Sidama and Oromia Regions.

**Study design and period**: This was a five-year retrospective study on the pattern and outcome of trauma patients who visited the Emergency Department of DURH from January 2015 to June 2019.

**Source population**: All trauma patients who visited DURH Emergency Department

**Study population**: All trauma patients during the past five years visiting DURH

**Inclusion criteria**: Injured patients' charts with complete clinical and socio-demographic information

**Exclusion criteria**: Patients who were referred to other centers after admission, non-traumatic patients visiting emergency department and patients' charts with missing variables.

**Sample size**: The required sample size was calculated using single proportion formula to obtain the sample size needed to estimate the prevalence injury. The Prevalence of injury was taken from the previous study conducted in the region, P = 0.494 (8), confidence interval = 95% and margin of error (d) = 5%. Hence, the required sample size was 384 injury patients visiting the surgical Emergency Department.

**Sampling technique**: All patients with injury within the study period were identified (N=4390), and this gave us the sampling frame. The required sample size was obtained by systematic random sampling technique with a skip interval (K=11). Finally, 376 patients were included in the study while the rest eight cases were excluded due to incomplete records.

A structured questionnaire adapted from WHO injury surveillance and validated for low and middle-income countries was used to collect the information. We included in the questionnaire the following: socio-demographic data (age, sex, level of education, place of residence, income based on GNI per capita, living condition, and occupation), injury mechanism, interval time from injury to admission, systolic blood pressure (BP), diastolic blood pressure (DB), pulse rate (PR), respiratory rate (RR), type, mechanism and pattern of injury, revised trauma score, Glasgow Coma Score (GCS) and length of stay (LOS). The outcome was status on discharge.

Patient status on discharge from hospital were dependednt variable while sociodemographic characteristics including age, gender, residence, educational status, income and living condition; patient clinical parameters such as blood pressure, GCS, RTS, respiratory rate, heart rate, injury characteristics and triage including types of injury, mechanism of injury, mode of transportation, time of arrival to institution, hospitalization, surgery, ICU admission/duration, mechanical ventilation are independent variables.

**Data quality assurance**: The structured questionnaire was prepared in English first and translated into the local language, Amharic and again back into translation to English was made to ensure the consistency of the questionnaire. Pretest was done on 5% of the sample size. Data collectors and supervisors were trained on each item included in the study tools, objective of the study and relevance of study. During data collection, regular supervision and follow-up were made. The investigator cross-checked for completeness and consistency of data on a daily basis.

**Statistical analysis and processing**: After completeness of the data was crosschecked manually, it was entered into epi info version 7 computer programs and transported to Statistical Package for the Social Sciences (SPSS) version 22-computer program for further analysis and cleaning. Descriptive statistics were used to summarize study findings. Continuous variable was described by mean ± standard deviation. Proportion and frequency table was used to summarize categorical variable. The outlier of the data was checked using standardized residual tests, and multi-collinearity for continuous data was checked by variance inflation factors (VIF) and tolerance. Linearity of the continuous variables with respect to the logit of the dependent variable was assessed via the Box-Tidwell procedure, and all continuous independent variables were found to be linearly related to the logit of the dependent variable. Association of trauma injury related variables and demographic characteristics with the outcome of a patient on discharge were analyzed using chi-square, fisher's exact test and binary logistic regression with odds ratio and 95% CI in the univariate analysis. Multivariate logistic regression analysis was used to determine the association of combined risk factors for patient outcome on discharge (died or improved). All variables with a p < 0.20 in the univariate analysis were entered into the logistic regression model. Odds ratios (OR) and 95% confidence intervals were then calculated. A pvalue less than 0.05 was considered significant. Additionally, continuous trauma related variables were checked for association with dependent variable by area under the curve of a Receiving Operating Curve (ROC) analysis with a confidence interval of 95% and p-value.

**Ethical considerations**: Ethical clearance and approval was obtained from Ethical Review Board (IRB). As the study was retrospective, the IRB waived that the research could be done based on record review without contacting the patients. A support letter was obtained from the Medical Director's office of the hospital for retrieving retrospective data from the database and records. All the information was kept confidential, and no individual identifiers were collected.

## Results

A total of 9420 cases were received from the Surgical Emergency Department of Dilla University Teaching and Referral Hospital from January 2008 – June 2019, from which 4390 patients were trauma cases. Three hundred seventy six cases had complete documentation from 384 included samples giving a response rate of 98%.

**Socio-demographic characteristics**: The mean and SD of ages of study participants was 24.5(±10.3). In this study, the youngest and the oldest trauma victims were 3 years and 66 years old respectively. The majority of the victims were in the age range of 20–40 years, 196(52.1%) whereas 29(7.7%) cases were in the age range of greater than 40 years old. The majority of the cases experiencing trauma were rural dwellers 225(59.8%) compared to urban dwellers who were 151(40.2%). In this study, patients with low socioeconomic status accounted for the majority of trauma incidents compared to middle and high socio-economic status. More than half of the study participants were single, 243(62.2%), whereas there was only one divorced trauma victim. On the other hand, students accounted for the majority of trauma incidents, 161(42.8%), followed by farmers, 80(22.1%).

**Figure 1:**
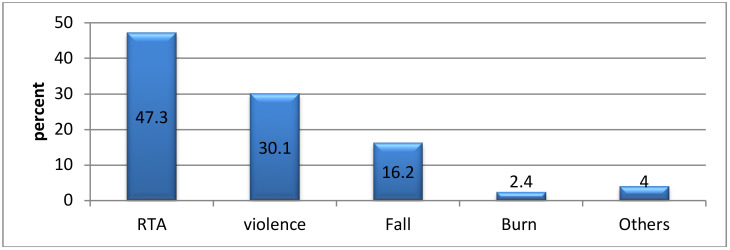
Mechanism of injury

**Epidemiology of trauma**: In this study, the overall prevalence of trauma was 46.6%. Road traffic accident was the commonest mechanism of injury, 178(47.3), followed by assault, 113(30.1%), whereas burn injury was the lowest mechanism of injury, 22 (5.9), (Figure 1).

**Figure 2:**
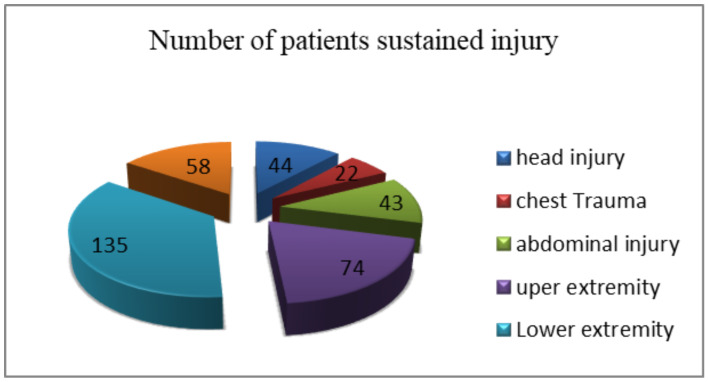
Types of injury

The commonest type of injury was lower extremity injury, 135(35.9%), followed by upper extremity, 74(19.7), and polytrauma, 58(15.4), whereas chest trauma was found to be the lowest type of injury (Figure 2).

**Outcomes of trauma**: In this study, there was 23(6%) deaths from included samples visiting Surgical Emergency Department. Mortality of cases were more prevalent in the 20–40 age group, 13(56.5%), and among males, 12(52.2%). There were more deaths among rural dwellers 16(69.6%) compared to the ones who lived in urban areas, 7(30.4). The majority of deaths occurred among students, 6(20.1%), and males, 12(52.2). Besides, the mortality of the cases were very high in low socio-economic status when compared to those with middle and high socio-economic status (Table 1).

**Table 1:** Descriptive statistics on sociodemographic characteristics and outcomes of patients visiting surgical emergency department of Dilla university referral hospital from January 2015 to June 2019

Variable	Category	Outcome		Number (%)
		Improved	Died	
Age	<20	145	6	151(40.2)
	20–40	183	13	196(52.1)
	>40	25	4	29(7.7)
Gender	Male	255	12	267(71)
	Female	98	11	109(29)
Place of residence	Urban	144	7	151(40.2)
	Rural	209	16	225(59.8)
Educational status	Illiterate	66	10	76(20.2)
	Read and write	118	5	123(32.7)
	Primary school	69	4	73(19.4)
	Secondary school	90	4	94(25)
	College and above	10	0	10(2.7)
Marital status	Single	223	13	236(62.8)
	Married	125	10	135(35.9)
	Widowed	4	0	4(1.1)
	Divorced	1	0	1(0.3)
Occupation	Student	155	6	161(42.8)
	Civil servant	10	1	11(2.9)
	Driver	34	1	35(9.3)
	Farmer	75	8	83(22.1)
	Unemployed	73	7	80(21.3)
	Others	6	0	6(1.6)
Income	Low	264	16	280(74.4)
	Medium	87	3	90(24)
	High	2	4	6(1.6)
Living condition	Home	351	22	373(99.2)
	Street	2	1	3(0.8)

Road traffic accident was the commonest cause of death, 12(52.2%), followed by assault, 8(34%), and the least was found in burn patients. Polytrauma was responsible for the majority of deaths, 10(43.5%), followed by head injury, 9(39.1). The mortality of the cases with lower trauma score at admission were very high. The mortality of cases that arrived in health institution after one hour was very high (Table 2).

**Table 2:** Cause and outcomes of trauma patients visiting surgical emergency department of Dilla university referral hospital from January 2015 to June 2019

Variable	Category	Outcome		Number (%)
			
		Improved	Died	
**Mechanism of injury**	RTA	166	12	178(47.3)
	Assault	105	8	113(30.1)
	Fall	58	3	61(16.2)
	Burn	9	0	9(2.4)
	Others	15	0	15(4)
**Types of injury**	Head	35	9	44(11.7)
	Chest	21	1	22(5.9)
	Abdomen	42	1	43(11.4)
	Upper extremities	74	0	74(19.7)
	Lower extremities	133	2	135(35.9)
	Polytrauma	48	10	58(15.4)
**Revised trauma score**	<10	10	10	20(5.3)
	≥10	343	13	356(94.7)
**Glasgow Coma scale**	Mild	349	14	363(96.5)
	Moderate	4	2	6(1.6)
	Severe	0	7	7(1.9)
**Time to arrive institution**	Immediate(<1hr)	162	2	164(43.6)
	Within hrs(1–24hrs)	190	13	203(54)
	Within days(>24hrs)	1	8	9(2.4)
**Mode of transportation**	Ambulance	27	7	34(9)
	Bajaj	141	6	147(39.1)
	Taxi	169	8	177(47.1)
	Others	16	2	18(47.8)
**Operated on**	Yes	10	5	15(4)
	No	343	18	361(96)
**Hospitalization**	Less than one day	1	0	1(0.3)
	1–7days	329	11	340(90.4)
	>7days	23	12	35(9.3)

**Figure 3:**
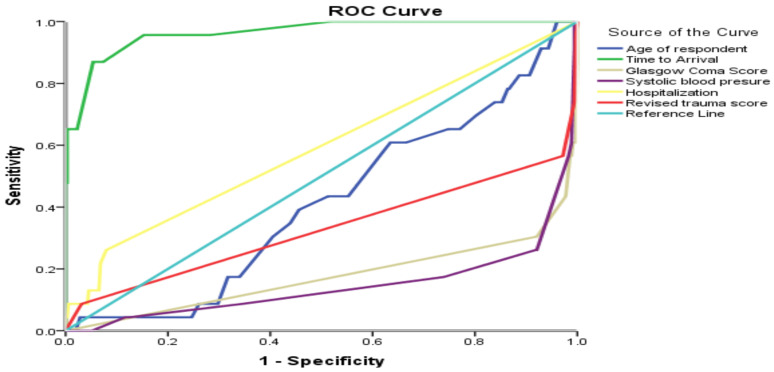
the receiver operating characteristic curve of age, GCS, RTS, SBP, time to arrival and Hospitalization

**Determinants of patient mortality after injury**: Using ROC curve, we assessed the individual performance of age of respondents, Glasgow coma scale, revised trauma score, time of arrival, systolic blood pressure and length of hospitalization to predict trauma outcome. In assessing the acceptable discrimination for predicting trauma injury outcome, both time of arrival and length of hospitalization were above the reference line, and the other variables like Glasgow coma scale, revised trauma score and systolic blood pressure were below the reference line. The ROC analysis curve yielded an area under cure (AUC) =0.96, 95% CI: 0.92–1.00 for time of arrival which showed the highest predictive ability and was excellent at discrimination of trauma patient mortality. The AUC for the length of hospitalization was 0.59, 95% CI: 0.46–0.72, which is poor at discrimination of patient outcome (Figure 3).

Bivariable logistic regression with crude odds ratio on various clinical characteristics in injury patients and potentially confounding factors that affected the relationship between primary predictor variables and a dichotomous categorical outcome (dead or improved) were considered, with a 95% confidence interval and P < 0.05. Patients with associated abdominal injury, lower extremity injury, time to arrival > 24hrs, being admitted for 1–7 days in the hospital, revised trauma score <10, decrease in GCS and operation had higher odds of mortality on bivariate analysis.

Sex, income, age group, type of injury, time of arrival, revised trauma score (RTS), operated on, and Glasgow coma scale (GCS) were the variables that had p-value <0.2 and were considered for multiple logistic regression analysis. After adjustment for multiple logistic regression, the mortality after an injury is three times more likely in a patient with a revised trauma score of less than ten (AOR] =2.5, 95% CI= [1.8, 25.6]). One unit in decreasing GCS was associated with three times [AOR] =0.3, 95% CI= [0.13, 0.5]) higher odds of predicting mortality. Being admitted for 1–7 days in hospital was 90% [AOR] =0.1, 95% CI= [0.01, 0.8] less likely to predict mortality compared to those admitted for >7 days. Being a middle income was less likely to develop hospital mortality than low income category. With regard to time to admission, those with time of arrival <1hr and 1–24hrs were less likely to have hospital mortality compared to those whose time of arrival was >24hrs (Table 3).

**Table 3:** Bivariable and multivariable logistic regression for factors associated with injury outcomes

Variable	Category	Outcome		COR	P-value	AOR	P-value
						
		Improved	Died				
Age	<20	145	6	0.26(0.70–0.98)	0.04	1.87(0.09–39)	0.68
	20–40	183	13	0.44(0.13–1.47)	0.18	0.51(0.25–10)	0.66
	>40	25	4	1		1	
Gender	Male	255	12	0.42(0.18–0.98)	0.04	0.54(0.08–3.8)	0.54
	Female	98	11	1		1	
Income	Low	264	16	0.03(0.01–0.18)	<0.001	0.09(0.002–3.5)	0.2
	Medium	**87**	**3**	**0.02(0.002–0.13)**	**<0.001**	**0.003(0–0.4)**	**0.02**
	High	2	4	1		1	
Time to arrival	Immediate(<1hr)	**162**	**2**	**0.002(0–0.02**	**<0.001**	**0(0–0.011)**	**<0.001**
	Within hrs(1–24hrs)	**190**	**13**	**0.009(0.001–0.07)**	**<0.001**	**0(0–0.016)**	**<0.001**
	Within days(>24hrs)	1	8	1		1	
Types of injury	Head	35	9	1.23(0.45–3.35)	0.68	1(0.09–10.5)	0.99
	Chest	21	1	0.22(0.02–1.9)	0.17	0.28(0.01–8.7)	0.46
	Abdomen	42	1	0.11(0.01–0.9)	0.04	0.35(0.02–5)	0.44
	Upper extremities	74	0	-	-	-	-
	Lower extremities	133	2	0.07(0.02–0.34)	0.001	0.07(0.002–2)	0.12
	Polytrauma	48	10	1		1	
Operated on	Yes	10	5	9.53(2.0–30.8)	0.0002	2(0.06–60)	0.69
	No	343	18	1		1	
Hospitalization	Less than one day	1	0	-	-	-	-
	1–7days	**329**	**11**	**0.06(0.03–0.16)**	**<0.0001**	**0.1(0.01–0.8)**	**0.038**
	>7days	23	12	1		1	
RTS	<10	**10**	**10**	**26.4(9.35–74.41)**	**<0.0001**	**2.5 (1.8–25.6)**	**0.04**
	≥10	343	13	1		1	
GCS Median(IQR)	15(0)			**0.45(0.34–0.58**	**<0.001**	**0.3(0.13–0.5)**	**0.001**

COR: Crude Odd Ratio, AOR: Adjusted Odd Ratio, GCS: Glasgow Coma Scale, RTS: Revised Trauma Score, IQR: Interquartile range

## Discussion

The prevalence of injury in this study was 46.6%, which is comparable with a study conducted in Yirgalem (49%)(2). However, it was higher than studies conducted in the University of Gondar (25%)(7) and Tikur Anbesa Hospital (32%)(22). This discrepancy might be due to poor road safety and more motorcycle transportation in our study area. It was lower than a study conducted in Amhara Regional State (55.6%)(6) and Jimma University Referral Hospital (5), and this may be explained by study setting and large sources of population compared to this study area. The prevalence of injury in one of Nigerian Tertiary Hospitals was lower than half of ours, and this might be due to better mode of transportation and road safety (23).

This study revealed that male gender and young age group, 20–40 age group, were the most commonly injured ones who are supposed to be the gear changers of the economic activities of the country. This finding is consistent with findings of WHO injury and violence surveillance facts and other studies conducted in sub-Saharan African countries (10,19–21). The majority of the patients were primary school attendants and students. This finding is in line with the study conducted in Yirgalem, Nigeria, and Tanzania (2,23,24). A multicenter study conducted in Amhara Regional State found that the illiterate and farmers were the most likely injured patients unlike the findings of this study (6).

In this study, patients from rural area are more affected with injury as compared to urban areas and this finding is consistent with a study done in the University of Gondar and Yirgalem (2,7). This is explained by more hazardous type of occupation and other activities in rural areas. However, patients residing in urban areas were affected more compared to patients from rural settings in multicenter study conducted in Amhara Regional State and in sub-Saharan African countries (6,20). This discrepancy might be due involvement of multicenter from big towns in the Amhara region and sub-saharan countries.

Road traffic accident was the commonest type of injury, (47.3%), followed by interpersonal violence, (30.1%), which is comparable with findings of sub-Saharan African countries and WHO reports (1,20,21,25,26). In this study, lower extremity injury was the most common type of injury unlike other studies conducted in Africa which revealed prevalence of head injury (5–8,22,27,28). This discrepancy might be due to head injury case referral as our hospital did not have neurosurgical facilities.

The study showed that the prevalence of death was high (6%) compared to a similar study conducted in the University of Gondar (2.11%). Which is relatively lower by more than half and the difference might be explained by study setting, referral linkage, and admission.

This study showed that patients who arrived after 24 hours' injury were more likely to die compared to patients who arrived within 24 hours. This finding is consistent with studies conducted in Tanzania (24) and Ruanda (8), which showed that those who arrived after 24 hours were four times more likely to die. This can be due lack of healthcare facility nearby since most of our study participants were from rural areas. This study revealed that the duration of hospitalization between 1–7 days were 90% less likely to predict mortality compared to patients hospitalized for greater than 7 days, which was an independent predictor of hospital mortality. This finding is in line with a study in Tanzania in which hospitalization of greater than 7 days was two times more likely to die (24).

Our study showed that Revised Trauma Score and Glasgow Coma scale was significantly associated with patient mortality. The patient mortality increases three times in reduction of one unit Glasgow Coma scale whereas patient mortality was three times more likely in patients with revised trauma score of less than 10. This explains that quick assessment of injury severity can significantly affect the outcome of trauma patients. Application of this scoring system helps in categorizing high risk patients in need of early intervention, which leads to better outcome.

In conclusion, the prevalence of injury in this study was very high and took the lives of the most productive age groups of the population. Road traffic injury and violence were the two most common causes of injury, which counted for more than 80% of deaths (20/23). Patients with low trauma score and those who came late were more likely to have significant mortality and morbidity. In this study, there were more deaths associated with head injury and Polytrauma. There is a need to have urgent injury preventive and management strategies along with establishment of pre-hospital emergency medical service system.

This study has some limitations despite its significant contribution as a source of information for prevention and management strategies. As this is a cross-sectional hospital- based study, the findings are not generalizable to the general population. Besides, lack of trauma registry and incomplete patient charts were the major challenges.
